# Identification of a diagnostic model and molecular subtypes of major depressive disorder based on endoplasmic reticulum stress-related genes

**DOI:** 10.3389/fpsyt.2023.1168516

**Published:** 2023-08-15

**Authors:** Shuwen Huang, Yong Li, Jianying Shen, Wenna Liang, Candong Li

**Affiliations:** ^1^Research Base of Chinese Medicine Syndrome, Fujian University of Traditional Chinese Medicine, Fuzhou, Fujian, China; ^2^FuJian Key Laboratory of TCM Health State, Fuzhou, Fujian, China; ^3^LI Candong Qihuang Scholar Studio, Fujian University of Traditional Chinese Medicine, Fuzhou, Fujian, China

**Keywords:** major depressive disorder, endoplasmic reticulum stress, diagnostic model, molecular subtype, bioinformatics

## Abstract

**Subject:**

Major depressive disorder (MDD) negatively affects patients’ behaviours and daily lives. Due to the high heterogeneity and complex pathological features of MDD, its diagnosis remains challenging. Evidence suggests that endoplasmic reticulum stress (ERS) is involved in the pathogenesis of MDD; however, relevant diagnostic markers have not been well studied. This study aimed to screen for ERS genes with potential diagnostic value in MDD.

**Methods:**

Gene expression data on MDD samples were downloaded from the GEO database, and ERS-related genes were obtained from the GeneCards and MSigDB databases. Differentially expressed genes (DEGs) in MDD patients and healthy subjects were identified and then integrated with ERS genes. ERS diagnostic model and nomogram were developed based on biomarkers screened using the LASSO method. The diagnostic performance of this model was evaluated. ERS-associated subtypes were identified. CIBERSORT and GSEA were used to explore the differences between the different subtypes. Finally, WGCNA was performed to identify hub genes related to the subtypes.

**Results:**

A diagnostic model was developed based on seven ERS genes: KCNE1, PDIA4, STAU1, TMED4, MGST1, RCN1, and SHC1. The validation analysis showed that this model had a good diagnostic performance. KCNE1 expression was positively correlated with M0 macrophages and negatively correlated with resting CD4+ memory T cells. Two subtypes (SubA and SubB) were identified, and these two subtypes showed different ER score. The SubB group showed higher immune infiltration than the SubA group. Finally, NCF4, NCF2, CSF3R, and FPR2 were identified as hub genes associated with ERS molecular subtypes.

**Conclusion:**

Our current study provides novel diagnostic biomarkers for MDD from an ERS perspective, and these findings further facilitate the use of precision medicine in MDD.

## Highlights

1. A diagnostic model based on ERS-related genes was developed.
2. This model had good diagnostic performance for MDD.
3. KCNE1 correlated with M0 macrophages and resting CD4 memory T cells.
4. Two molecular subtypes with different ER scores and immune characteristics were identified.
5. NCF4, NCF2, CSF3R, and FPR2 were identified as the hub genes associated with subtypes.

## Introduction

Major depressive disorder (MDD) is a common mental disorder with an estimated annual prevalence of 4.4% worldwide, affecting more than 300 million people (1). It is ranked as the leading cause of disability globally and the third leading cause of the global burden of disease (2). MDD is chronic or recurrent in nature, usually associated with prolonged periods of depressed mood and anhedonia, and is accompanied by considerable morbidity, suicide risk, and mortality (3). Despite receiving evidence-based treatment, approximately 30–50% of patients remain unresponsive to therapy, imposing a huge economic burden on society (4). Depression is associated with many mental and physical disorders and is influenced by an interplay of genetic and environmental factors, suggesting that its underlying mechanisms are complex (5). Previous studies have suggested that the ineffectiveness of antidepressant drugs may be partly attributed to their failure to address important biological processes involved in the pathogenesis of depression (6). Therefore, it is essential to explore the molecular mechanisms underlying MDD and identify diagnostic markers and therapeutic targets for this disease.

The endoplasmic reticulum (ER) is the largest organelle in eukaryotic cells and is involved in the regulation of protein synthesis, folding, and transport (7). ER dysregulation can lead to the accumulation of unfolded and misfolded proteins in the lumen, stimulating the unfolded protein response, a process known as ER stress (ERS) (8). Gold et al. in 1988 proposed that MDD represents dysregulation of the stress system in a readily inducible stressful environment (9). Moreover, multiple studies have shown that ERS is involved in the pathophysiology of central nervous system disorders, such as schizophrenia and MDD (10, 11). Elevated ERS responses in the brain have been observed in many human and animal models of depression. For example, ERS-related proteins (such as GRP78, CHOP, and XBP1) were found to be associated with hippocampal damage and cognitive impairment in a rat model of stress, and the expression levels of ERS-related proteins (such as GRP78 and CHOP) in the hippocampus of patients with MDD were upregulated compared to those in control subjects (12, 13). Meanwhile, ERS has been proven to be associated with cardiovascular diseases as well as several chronic diseases, such as diabetes and inflammatory bowel disease (14). Notably, depression is connected with reduced heart rate variability, increased sympathetic nervous system, and platelet aggregation, all of which are risk factors for cardiovascular diseases (15). Hence, ERS is closely correlated with common comorbidities of depression. Besides, ERS plays a key role in mediating immune and metabolic responses, and these mechanisms also contribute to the development of psychiatric disorders such as depression (16). Taken together, ERS may be directly or indirectly involved in several key biological processes that alter the course of MDD. Currently, there is evidence that targeting ERS may be a new strategy for the treatment of this disorder (17). However, the specific biomarkers of the diagnosis of MDD have not been fully explored, especially from the perspective of ERS-related genes.

In this study, we collected the transcriptome data of patients with MDD and ERS-related genes from public databases and identified ERS-related differentially expressed genes (DEGs) between MDD and control samples. Next, genes with a diagnostic value for MDD were screened using LASSO analysis to establish a diagnostic model. The diagnostic performance of this model was assessed, and the correlation between diagnostic genes and immune infiltrates was analysed. Molecular subtypes were screened based on ERS-related diagnostic genes. Our findings may help further explore potential biomarkers of MDD and provide reference targets for drug development.

## Materials and methods

### Dataset acquisition and pre-processing

**Figure 1:**
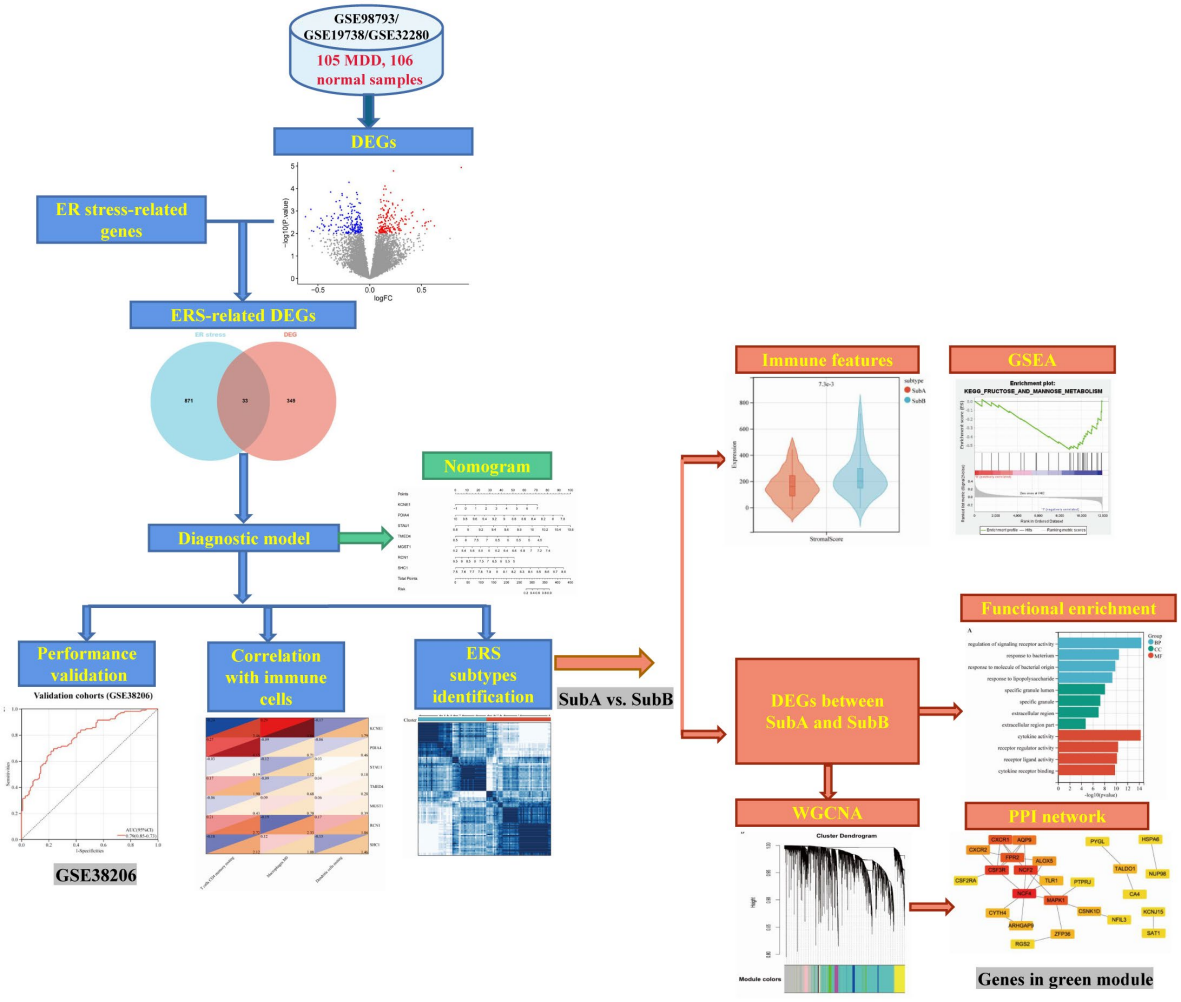
Workflow of this analysis.

The design of the analysis is illustrated in Figure 1. Raw gene expression profiles for four datasets (GSE98793 (18), GSE19738 (19), GSE32280 (20), and GSE38206 (21)) downloaded from GEO were included in this analysis. The sample information and detection platform for each dataset are presented in Table 1. A total of 114 patients with MDD and 115 healthy controls were analysed. GSE98793, GSE19738, and GSE32280 were selected as training cohorts. After merging the data from the three datasets, we used the sva package in R3.6.1^1^ to remove batch effects, and ultimately obtained the expression profile data of 211 samples, including 105 MDD and 106 control samples. The GSE38206 dataset was selected as the validation cohort. ERS-related genes were extracted from Genecards and the Molecular Signature Database v7.4 (MSigDB). The downloaded genes were duplicated and merged to obtain 904 genes.

**Table 1:** Specific information for four major depressive disorder-related datasets.

GO accession	Platform	Sample number		Sample source	Purpose
		Control	MDD		
GSE98793	GPL570	64	64	Whole blood	Training cohort
GSE19738	GPL6848	34	33	Whole blood	Training cohort
GSE32280	GPL570	8	8	Peripheral blood	Training cohort
GSE38206	GPL13607	9	9	Peripheral blood	Validation cohort

### Screening of ERS-related (DEGs in MDD patients vs. controls)

The Limma package (v 3.34.7) (22) was employed to perform differential expression analysis, and genes with value of *p* <0.01 were considered as DEGs between MDD and control samples. These DEGs were intersected with ERS-related genes to identify differentially expressed ERS-related genes. Correlations between ERS-related DEGs were calculated using the cor function^2^ in R.

### Establishment of protein–protein interaction (PPI) network

These ERS-related DEGs were imported into the STRING database (v 11.0) (23) to retrieve the interaction relationship between gene-encoded proteins, and PPI pairs with interaction scores ≥0.4 were retained to construct the PPI network.

### Univariate logistic regression analysis of ERS-related DEGs

Rms (v 6.3–0, https://cran.r-project.org/web/packages/rms/index.html) (24) in R was used to conduct univariate logistic regression analysis of ERS-related DEGs, and genes with value of *p* <0.05 were retained for further analyses.

### Development of a diagnostic model based on ERS-related genes

The LASSO algorithm in the R lars package (v 1.2) (25) was used to further screen the selected ERS-related DEGs. Next, RMS in R was used to perform multivariate logistic regression analysis, and the optimal ERS-related gene signature was selected as the diagnostic gene. The risk score (RS) was calculated based on the expression and coefficient of each diagnostic gene, followed by construction of a diagnostic model.

### Performance evaluation of diagnostic model

The receiver operating characteristic (ROC) curve method in the pROC package (v. 1.12.1) (26) was used to evaluate the efficacy of the diagnostic model constructed in the training and validation cohorts.

### Correlation analysis of diagnostic genes and immune states

In the training cohort, CIBERSORT was used to assess the proportion of immune cells in the samples, and the Kruskal–Wallis test in R was applied to compare the differences in the distribution of immune cells between the MDD and control groups. Meanwhile, the correlations between ERS-related diagnostic genes and immune cells with significant differences in MDD vs. control samples were calculated using the cor function in the R software.

### Construction and evaluation of the nomogram model

To predict the incidence of MDD, a diagnostic nomogram model was established using the rms package. Meanwhile, calibration curve and decision curve analysis (DCA) were employed to evaluate the prediction ability and practical utility of the model, respectively.

### Prediction of molecular subtype based on ERS-related genes

After extracting the expression levels of ERS-related diagnostic genes in the training cohort, ConsensusClusterPlus (v 1.54.0) (27) was used to determine the molecular subtype of the MDD samples. Furthermore, the ER score of each case was calculated using the GSVA (v 1.36.3) package, and the Kruskal–Wallis test was used to compare the differences in ER scores of different subtype groups.

### Comprehensive analysis of immune cells and molecules in different subgroups

CIBERSORT (28) was used to evaluate the distribution of immune cells in MDD samples in the training set, and the estimate package was then employed to calculate the ESTIMATE, immune, and stromal scores of patients with MDD. In addition, differences in immune cells and scores between different subgroups were analysed using the Kruskal–Wallis test.

We also compared the expression levels of immune checkpoint genes (CD27, CD274, and CD40) in different molecular subtypes using the Kruskal–Wallis test.

### Gene set enrichment analysis of different ERS subtypes

Based on the genome-wide expression levels in the training cohort, the GSEA database (29) was used to identify the KEGG signalling pathways that were significantly associated with the subtypes. We selected value of *p* <0.05 as the threshold for significant enrichment of the KEGG pathway.

### Screening of DEGs related to ERS-related molecular subtypes

Differential gene analysis was conducted on samples between subtypes, and genes with value of *p* <0.05 and |log_2_ Fold change (FC)| > 0.263 (also means FC > 1.2) were regarded as DEGs between different subtypes. To understand the biological functions of these genes, clusterProfiler was used to perform Gene Ontology (GO) (30) and KEGG pathway (31) enrichment analyses. A value of *p* <0.05 was defined as a significant enrichment result.

### Weighted gene co-expression network analysis

The MDD-related expression matrix was analysed using the R package WGCNA (v 1.6.1) (32) to identify highly covariant gene set modules. The specific analysis methods were as follows: first, the optimal power and connectivity k were selected to convert the gene expression matrix into a topological overlap matrix (TOM); second, the highly corrected genes were clustered into modules using clustering and dynamic pruning with these parameters (minModuleSize = 30 and mergeCutHeight = 0.25), and finally, the correlations between modules and molecular subtypes were calculated. The module with value of *p* <0.05 and with the highest connection with the subtype was selected for subsequent analysis. The hub genes in this module were screened using the gene significance (GS) and module membership (MM) indices; MM > 0.8 and GS > 0.2 were regarded as screening thresholds.

### Construction of PPI network based on hub genes

The STRING database was used to search for interactions between hub genes to build networks, and this network was visualised using Cytoscape (v 3.9.0) (33).

## Results

### Screening of 33 ERS-related DEGs between MDD and control samples

**Figure 2:**
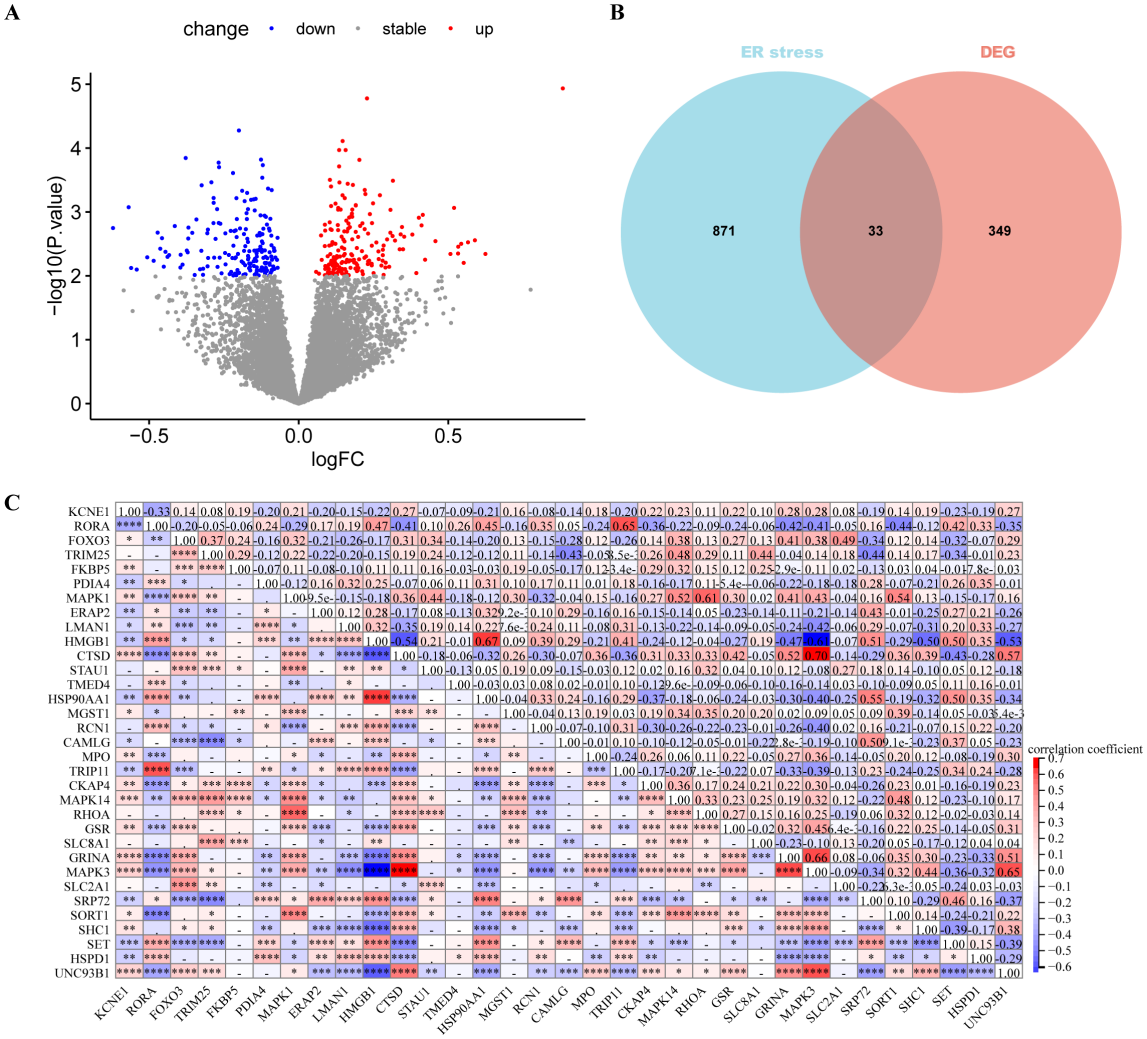
Differentially expressed gene (DEGs) analysis in major depressive disorder (MDD) patients. **(A)** Volcano plot of DEGs between MDD and control samples. Red and blue nodes represent upregulated and downregulated DEGs, respectively. **(B)** Venn plot showing the endoplasmic reticulum stress (ERS)-related DEGs (overlapping part). **(C)** Heat map revealing correlations between ERS-related DEGs. The numbers represent the correlation coefficients.

**Figure 3:**
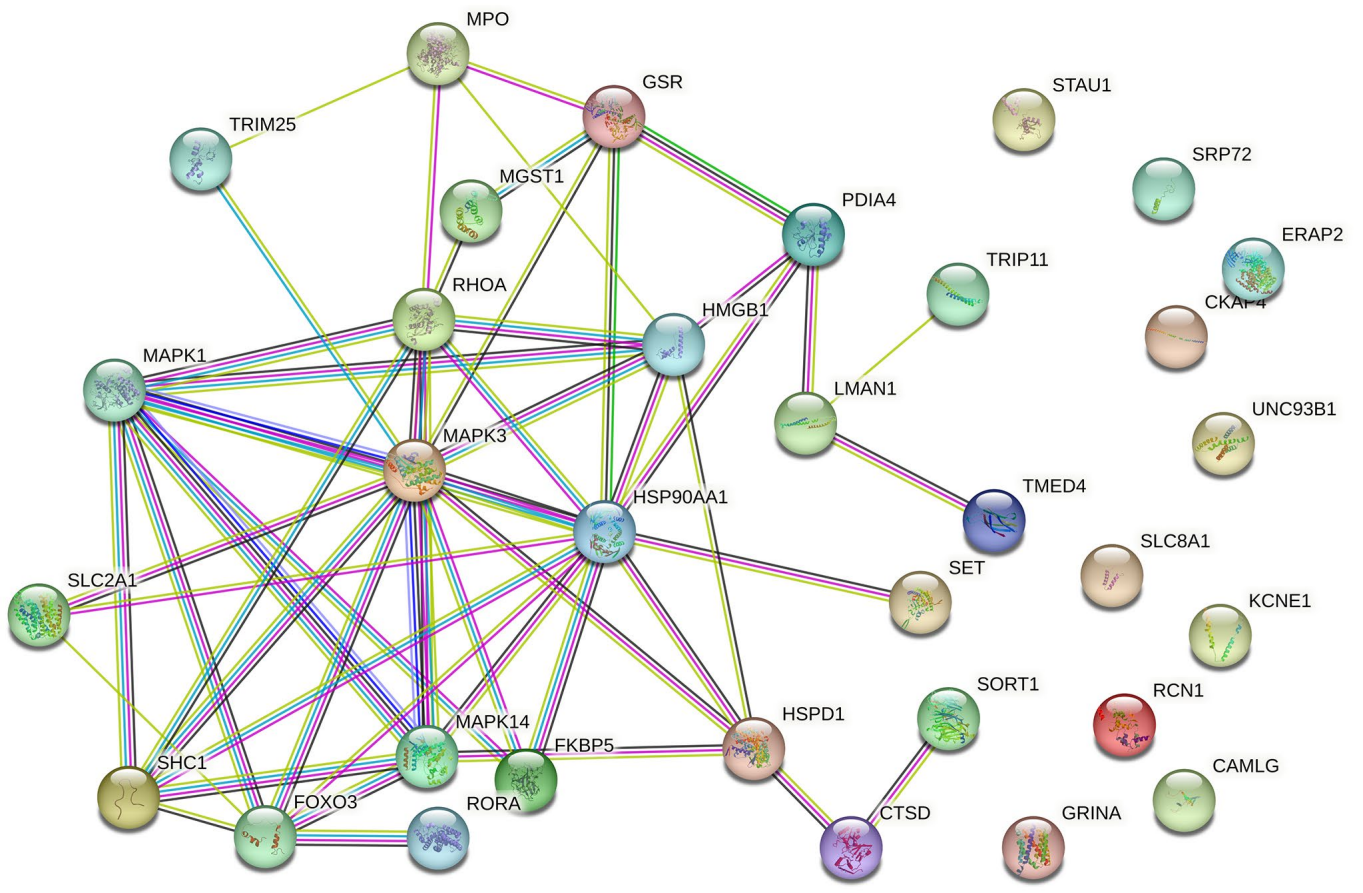
Protein–protein interaction (PPI) network of ERS-related DEGs.

Differential expression analysis revealed 382 DEGs (200 upregulated and 182 downregulated genes) between the MDD and control samples. The DEGs were visualised using a volcano plot (Figure 2A). After integration with the ERS-related genes, 33 ERS-related DEGs were identified (Figure 2B). We also observed a significant correlation between gene expression (Figure 2C). For example, MAPK3 was positively correlated with CTSD and negatively correlated with HMGB1. STRING was used to search for interactions between gene-encoded proteins and a PPI containing 23 ERS-related DEGs was established (Figure 3). We found that MAPK3 and MAPK1 were linked to a large number of genes.

### Construction of a diagnostic model based on seven ERS-related DEGs

**Figure 4:**
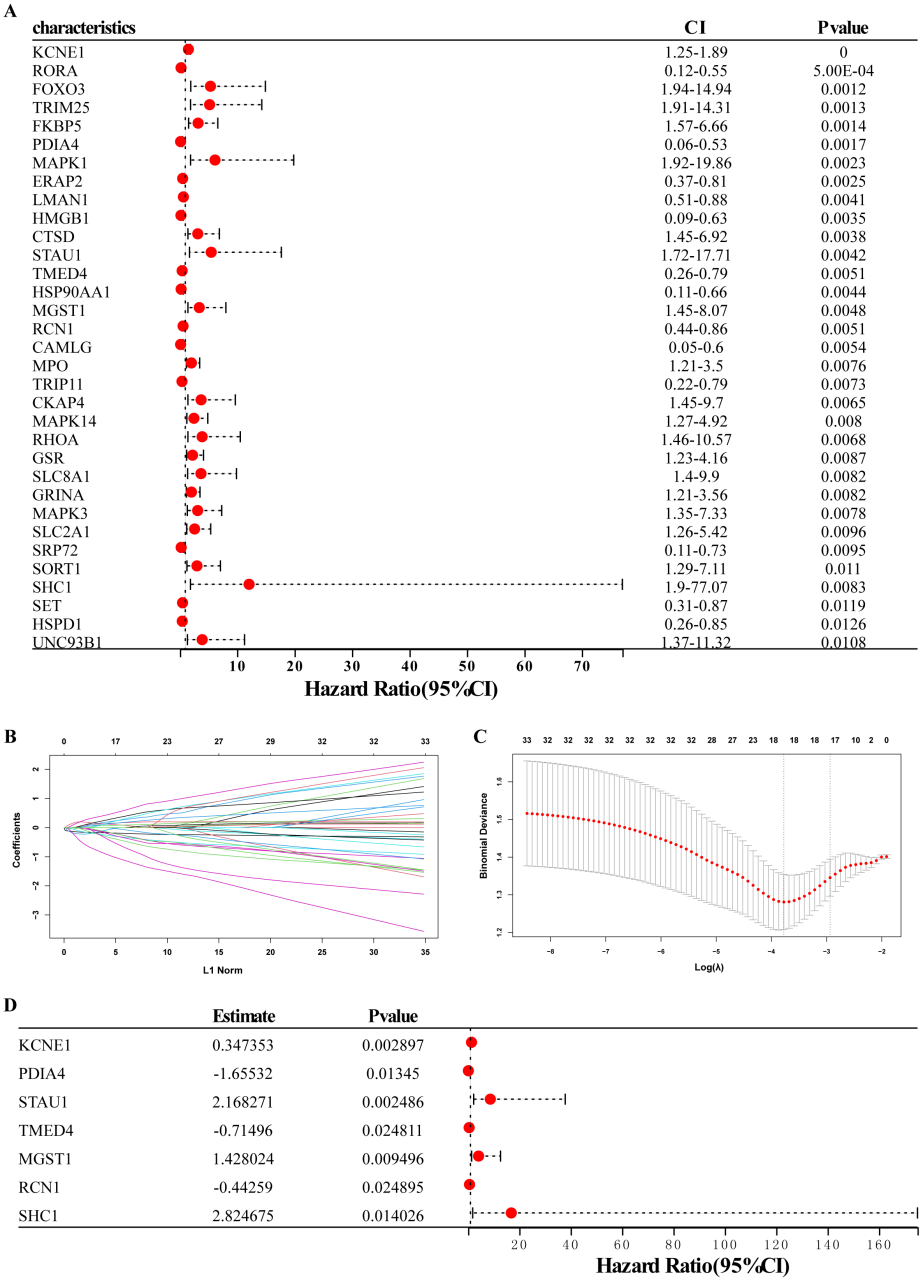
Establishment of the seven-gene signature diagnostic model. **(A)** Univariate logistic regression analysis confirmed that ERS-related genes were related to MDD occurrence. **(B,C)** LASSO regression analyses. **(D)** Multivariate regression analysis of seven diagnostic biomarkers for model construction.

Using univariate logistic regression analysis and LASSO regression algorithm, candidate ERS-related genes were screened from 33 ERS-related genes to predict the occurrence of MDD disease. These results indicate that the 18 genes could be used as potential diagnostic markers (Figures 4A–C). Multivariate regression analysis identified seven diagnostic biomarkers to construct the model (Figure 4D): KCNE1, PDIA4, STAU1, TMED4, MGST1, RCN1, and SHC1. RS values were calculated to establish diagnostic models based on the LASSO regression coefficients and the expression level of each gene.

### Diagnostic model had reliable predictive power for MDD in the training and validation cohorts

**Figure 5:**
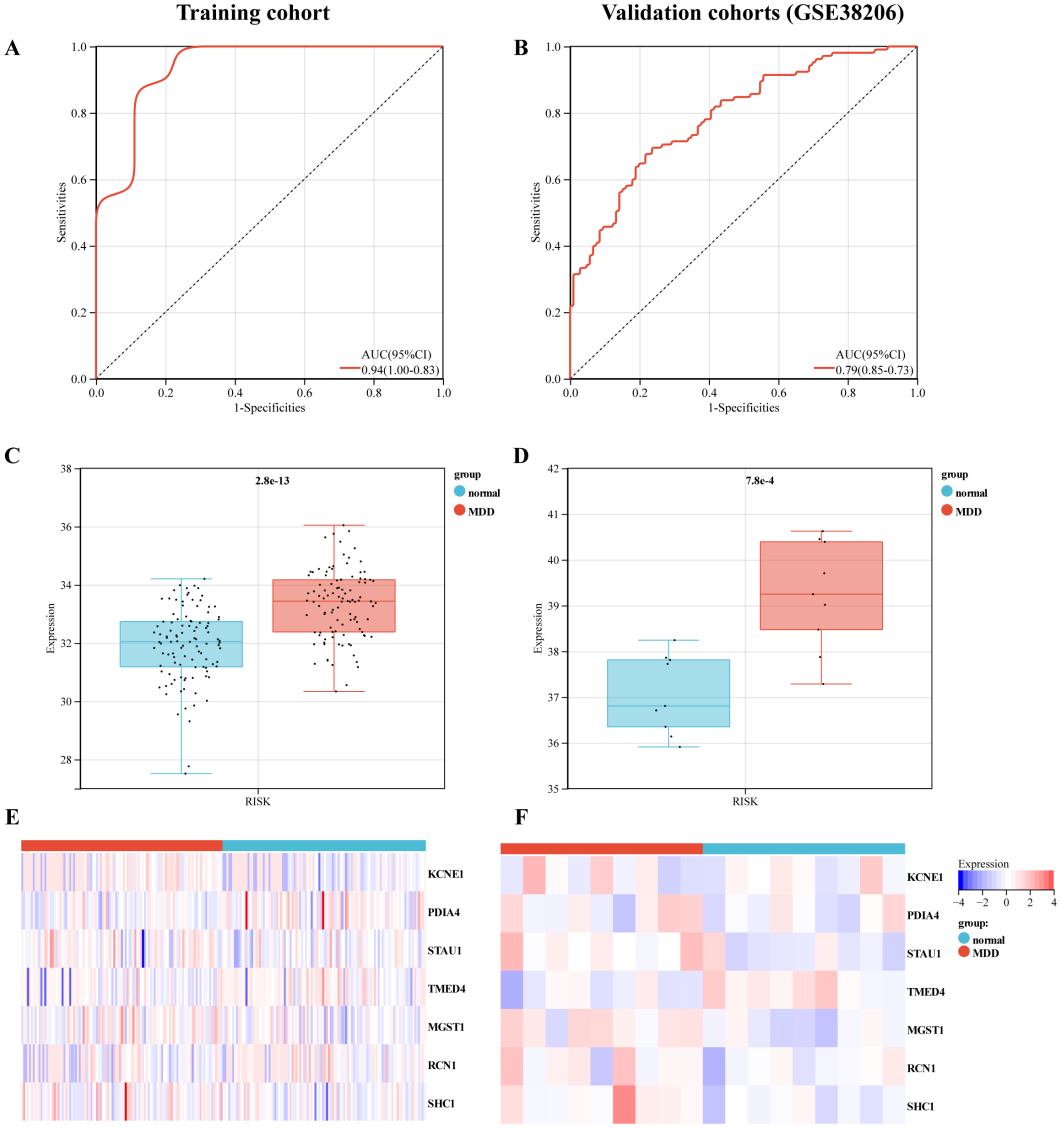
Evaluation of the predictive performance of diagnostic model in the training and validation cohorts. **(A)** Receiver-operating characteristic (ROC) curve of the model in the training cohort. **(B)** ROC curve of the model in the validation cohort (GSE38206). **(C)** Expression value of the risk score (RS) in the control and MDD groups within the training cohort. **(D)** RS expression values in the control and MDD groups within the validation cohort. **(E)** Heat map of the mRNA expression of seven ERS-related genes in control and MDD samples within the training cohort. **(F)** Heat map of the mRNA expression of seven ERS-related genes in the validation cohort.

Furthermore, we used training and validation cohorts to assess the predictive ability of the established model. The AUC values of ROC for models in the training and validation (GSE38206) cohorts were 0.79 (95% CI:0.73–0.85) and 0.94 (95% CI,0.83–1.00), indicating that this model had reliable predictive performance for MDD diagnosis (Figures 5A,B). The RS of MDD samples was significantly higher than that of control samples in both cohorts (Figures 5C,D). We also observed differences in the expression of these genes between the MDD and control groups in the training cohort (Figures 5E,F).

### Diagnostic nomogram model construction

**Figure 6:**
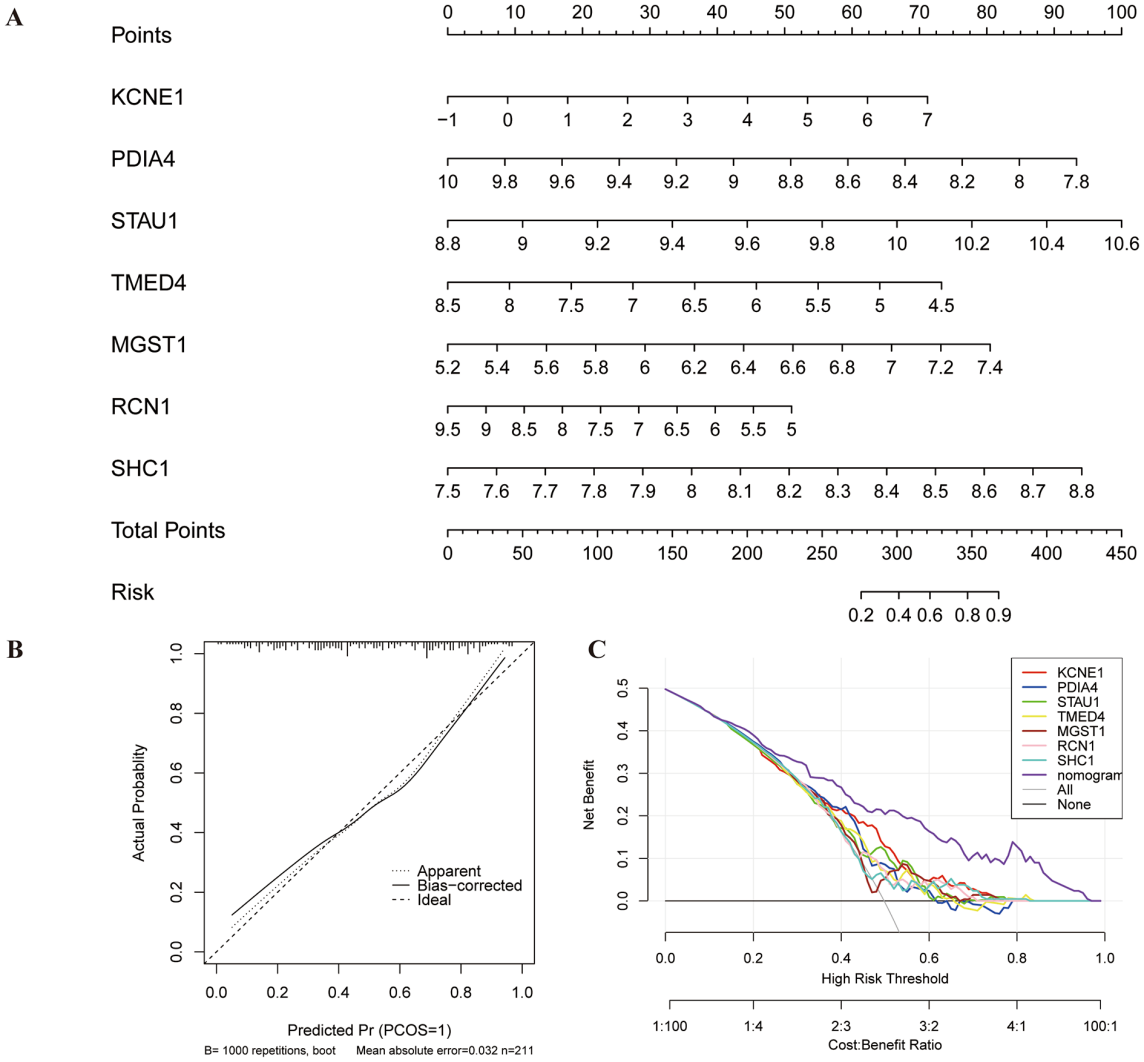
Construction of nomogram model for MDD diagnosis. **(A)** Nomogram based on seven genes to predict MDD risk. **(B)** Calibration curve to evaluate the diagnostic potential of the model. **(C)** DCA curve to assess the practical efficacy of the model.

A nomogram model based on seven biomarkers was generated to predict the risk of MDD. As shown in Figure 6A, each predictive marker was projected upward to the “point” of the value at the top of the nomogram to obtain a score of 0 to 100 points, and the total score of seven points was calculated to predict the probability of MDD risk. The calibration cure displayed that the predicted risk of MDD was in good agreement with the actual risk (Figure 6B). Moreover, the DCA revealed that the hub gene curves were above the grey line, indicating that the use of a nomogram to predict MDD risk had a significant net benefit (Figure 6C).

### Selected ERS-related DEGs were significantly associated with immune cell infiltration

**Figure 7:**
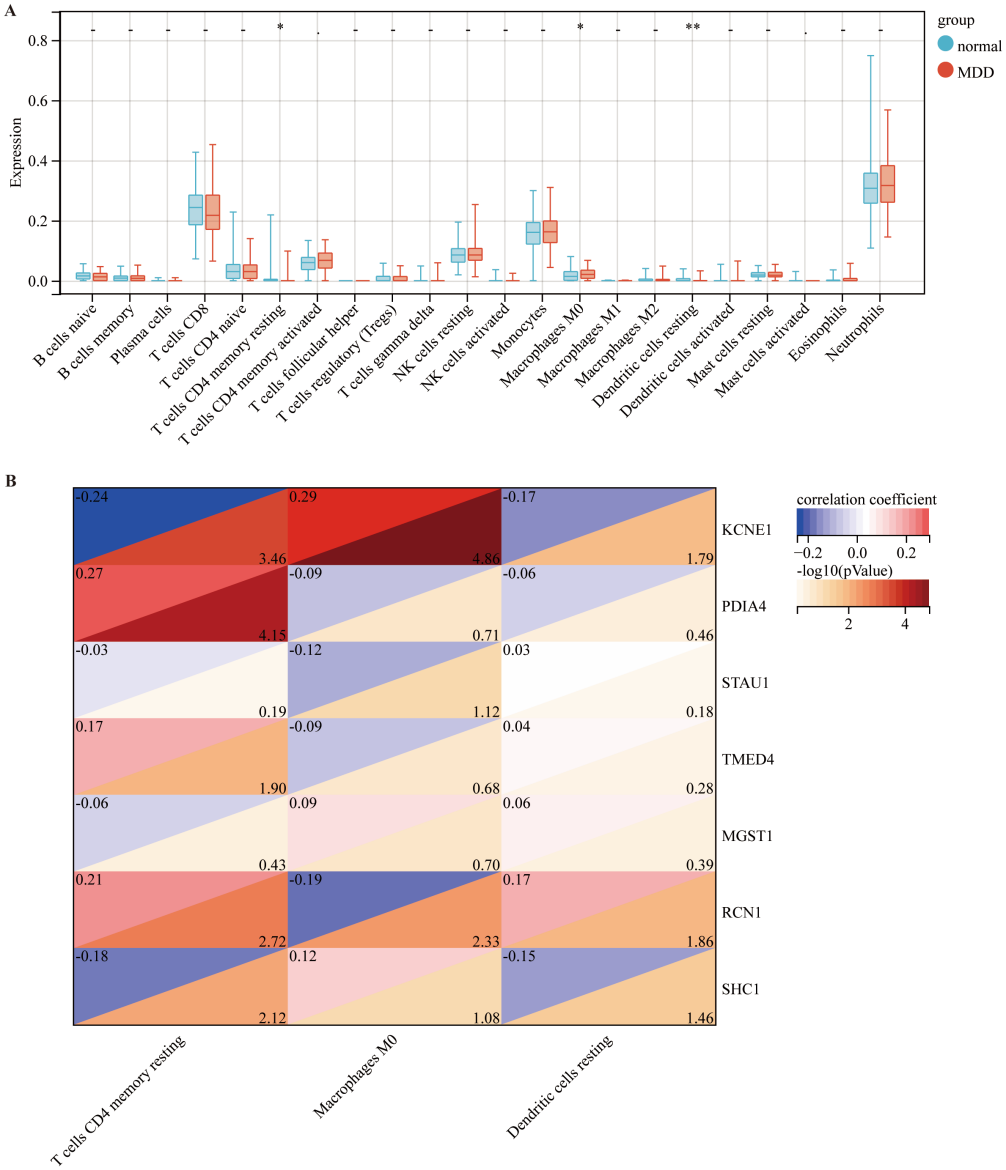
Comparison of immune cell infiltration amongst different disease groups. **(A)** Comparison of immune cell infiltration between control (blue box) and MDD (red box) groups. **(B)** Correlation of seven diagnostic markers with three types of immune cell infiltration in MDD.

To observe the immune statuses of the control and MDD groups, we compared the infiltration levels of immune cells between the two groups using CIBERSORT. The results indicated that the levels of infiltration of the three types of immune cells were significantly different between the two groups (Figure 7A). In brief, compared with the control samples, MDD samples had lower levels of resting CD4 memory *T* cells and resting dendritic cells, but had higher level of M0 macrophages. Next, the correlations between the seven diagnostic genes and the three immune cell types were analysed. KCNE1 expression showed the highest positive correlation with M0 macrophages (*r* = 0.29) and the highest negative correlation with resting CD4 memory T cells (*r* = −0.24) (Figure 7B).

### Two molecular subtypes of MDD patients identified using seven diagnostic markers

**Figure 8:**
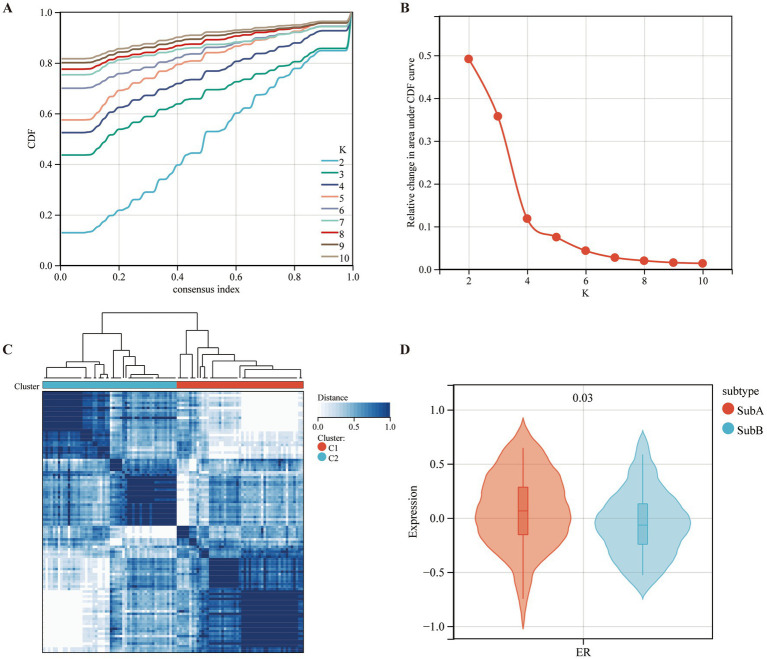
Identifying the ERS-based molecular subtypes in MDD patients. **(A)** Cumulative distribution function (CDF) curve of the consistency score. **(B)** Delta area plot of the relative change in the area under the CDF curve of the MDD samples. **(C)** The consensus score matrix for MDD samples indicates that the two clusters can be divided (*k* = 2). **(D)** Differences in ER scores between the two molecular subtypes.

Based on the expression levels of seven selected diagnostic markers, the molecular subtypes of MDD patients were screened. ConsensusClusterPlus software was used to calculate the optimal number of clusters. Based on the consistency matrix heatmap and CDF curve, k = 2 was defined as the optimal number (Figures 8A,B). Thus, two subtypes of patients with MDD were identified: SubA (*n* = 51) and SubB (*n* = 54) (Figure 8C). Next, the GSVA algorithm was used to assess the ER scores of each sample. The patients with MDD in the SubA group had significantly higher ER scores than those in the SubB group (Figure 8D).

### Differences in immune characteristics and immune checkpoint genes of the two ERS subtypes

**Figure 9:**
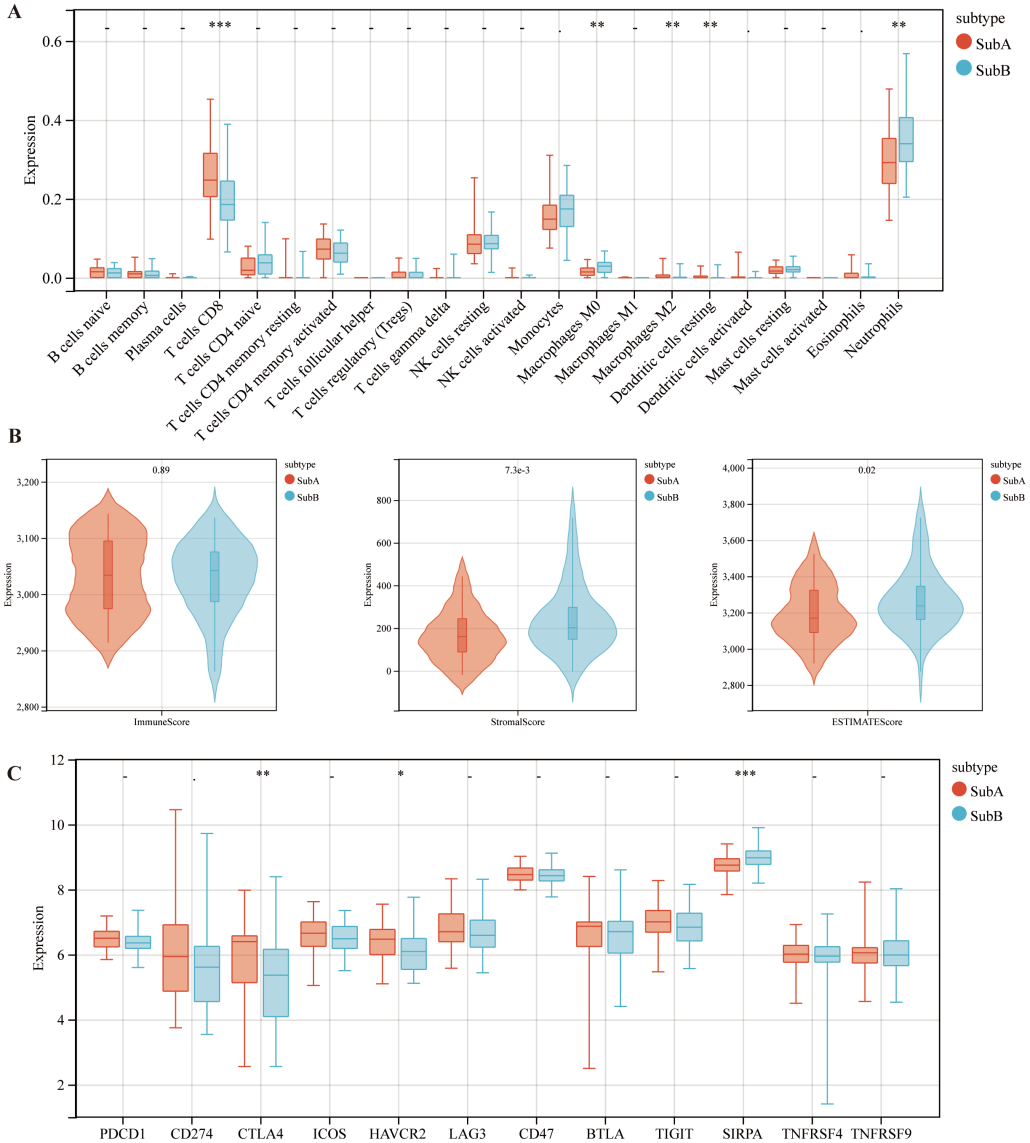
The distributions of immune cells and immune checkpoint genes between SubA and SubB subtypes. **(A)** Levels of immune cell infiltration between the two subtypes. **(B)** Differential distribution of immune features between the two subtypes. **(C)** Differential expression patterns of several immune checkpoint genes in the two subtypes.

Furthermore, the immune characteristics of the two subtypes were estimated using the CIBERSORT software. The infiltration levels of the five immune cell types differed between the two subtypes. Compared to the SubB type, the SubA type had significantly higher levels of CD8+ T cells, M2 macrophages, and resting dendritic cells, and significantly lower levels of M0 macrophages and neutrophils (Figure 9A). Meanwhile, SubB exhibited significantly higher stromal and ESTIMATE scores (Figure 9B), suggesting a higher degree of infiltration than that of SubA. We also observed that samples in the SubA group had higher expression of CTLA4 and HAVCR2 but lower expression of SIRPA than those in the SubB group (Figure 9C).

### GSEA revealing the significantly enriched biological pathways between two subtypes

**Figure 10:**
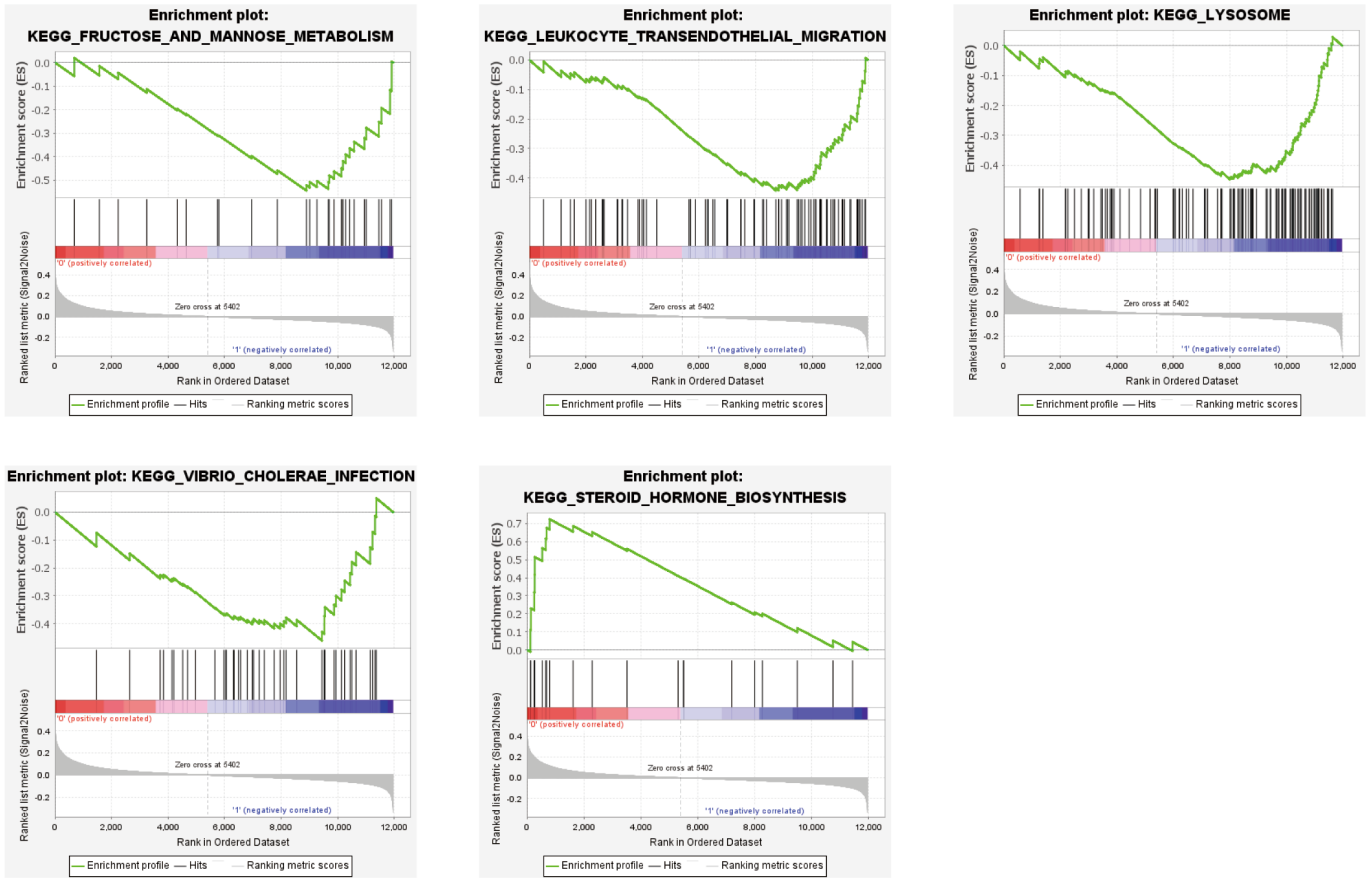
GSEA revealing the significantly enriched biological pathways between SubA and SubB subtypes.

GSEA was performed based on the two subtypes, and the top five significant biological pathways are shown in Figure 10. The results indicated that fructose and mannose metabolism, leukocyte transendothelial migration, lysosomes, and *Vibrio cholerae* infection were upregulated in the SubB group, and steroid hormone biosynthesis was upregulated in the SubA group.

### Differential expression analysis of two distinct subtypes

**Figure 11:**
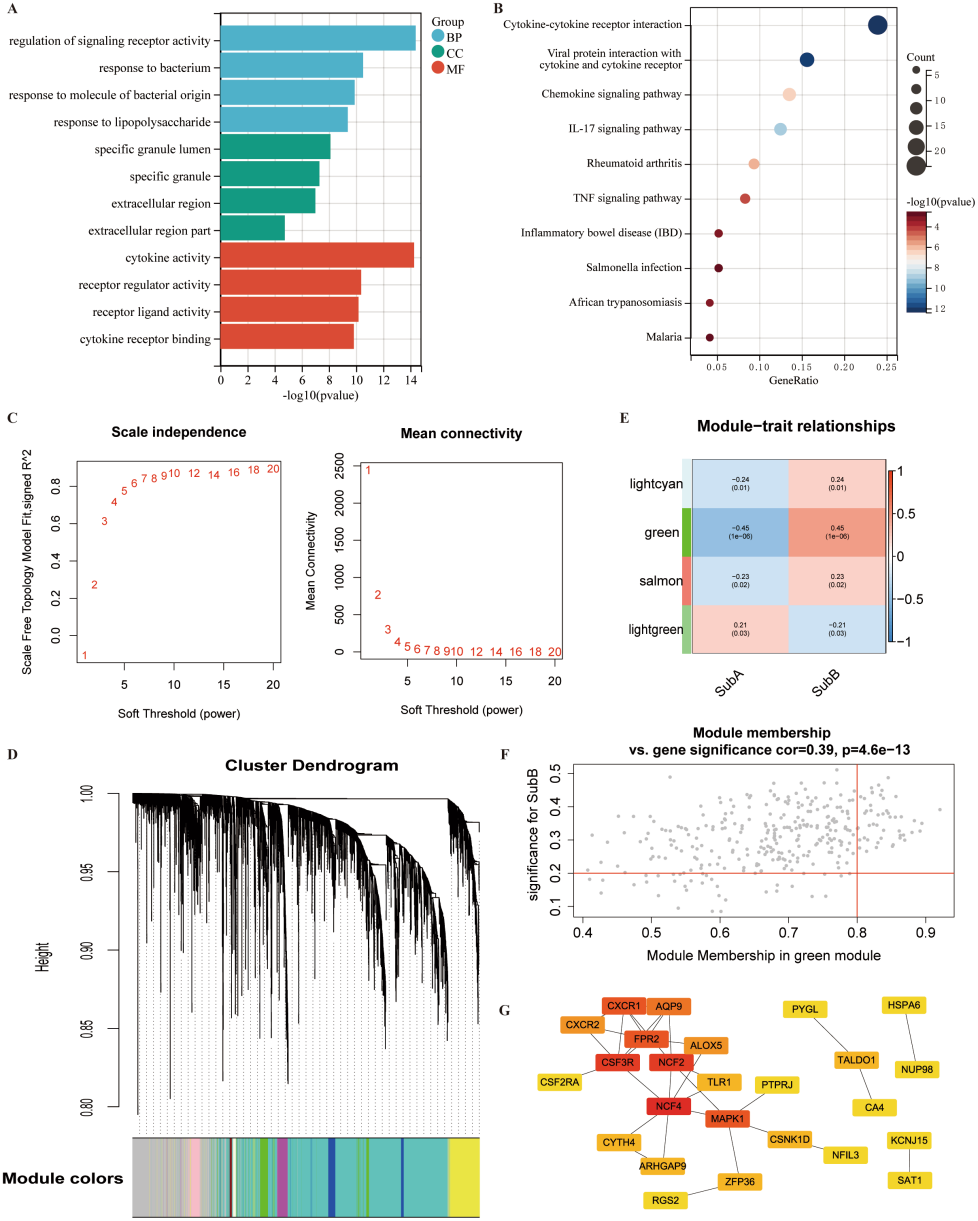
Identification of DEGs and hub genes associated with ERS subtypes. **(A)** Gene Ontology (GO) term analysis of DEGs between SubA and SubB subtypes. **(B)** KEGG pathway analysis of DEGs within the SubA vs. SubB groups. **(C)** Analysis of scale-free indices and mean connectivity for various soft-threshold powers. **(D)** Cluster dendrogram developed using weighted correlation coefficients. Each colour represents a module. **(E)** Correlation between modules and ERS subtypes. The upper numbers indicate the correlation coefficients and lower numbers indicate the value of *p*. **(F)** Scatter plot of correlations between gene significance (GS) and module membership (MM). **(G)** PPI network of the hub genes in the green module. Red indicates the nodes with high connectivity.

A total of 1,054 DEGs, including 468 upregulated and 586 downregulated genes, were identified between SubA and SubB subtypes. To explore the biological function of these DEGs, functional enrichment analysis was conducted. DEGs were mainly enriched in 977 GO terms (Figure 11A), such as regulation of signalling receptor activity (BP), specific granules (CC), and cytokine activity (MF); they were significantly involved in 27 KEGG pathways, such as the TNF signalling pathway and inflammatory bowel disease (Figure 11B).

### Identification of hub genes for ERS subtypes using WGCNA

The identified DEGs were selected for WGCNA to screen hub genes. Based on the scale free value and mean connectivity index, the optimal soft threshold was β = 9 when correlation coefficient > 0.85 (Figure 11C). In total, 85 modules were merged (Figure 11D). Next, the correlation between module genes and ERS subtypes was analysed, and the green module exhibited the most significant positive correlation with the SubB subtype (*r* = 0.45, value of *p* <0.05) (Figure 11E). A total of 320 genes in the green module served as potential key genes related to these subtypes. Further, genes with MM > 0.8 and GS > 0.2 were considered as hub genes, and 48 genes were obtained (Figure 11F).

To explore the interactions between these 48 hub genes, a PPI network was constructed. A total of 25 genes were included in this network (Figure 11G). Several nodes with high connectivity were identified, including NFC4, CSF3R, NCF2, and MAPK.

## Discussion

MDD is a highly debilitating disorder that affects millions of people worldwide and places a burden on families and communities (34). Given the high heterogeneity and complex pathological features of MDD, its diagnosis remains challenging. Current diagnosis mainly relies on the clinical assessment of patients’ self-reported symptoms and lacks objective tests; therefore, MDD still requires the use of specific markers for a definitive diagnosis (35). In addition, evidence suggests that high rates of misdiagnosis may contribute to poor recovery in patients with MDD due to limited knowledge of the diagnostic markers of the disease (36). Therefore, there is an urgent need to develop reliable detection methods for clinical practise. Previous evidence points to the involvement of ERS in the pathogenesis of MDD; however, few relevant diagnostic markers have been identified. This study aimed to screen for ERS genes associated with MDD and explore their potential diagnostic value. In this study, we developed a diagnostic model based on seven ERS-related genes. The validation results indicated that it had good diagnostic performance and closely correlated with the level of immune cell infiltration. Furthermore, two molecular subtypes associated with ERS were identified that differed significantly in terms of ER scores and immune characteristics.

Seven genes were included in the diagnostic model: KCNE1, PDIA4, STAU1, TMED4, MGST1, RCN1 and SHC1. The product of Potassium Voltage-Gated Channel Subfamily E Regulatory Subunit 1 (KCNE1) belongs to the KCNE family and modulates the function of voltage-gated K(+) channels (37). Previous research has shown that KCNE1 regulates neuronal K(+) channels and resting membrane potential (38). The role of KCNE1 in MDD has not been extensively studied, with only McCaffery et al. proposing that KCNE1 is associated with changes in depressive symptoms over the course of a year (39). Notably, the contribution of K(+) channels is mainly due to the expression of the KCNE, KCNQ, and ERG isoforms (40). Amongst these, the modulators (retigabine) of KCNQ channels have been shown to improve depressive symptoms and have the potential to treat MDD (41). Based on these findings, we speculate that KCNE1 may also be involved in the neurophysiological mechanisms of stress recovery by modulating neuronal activity, thus presenting a therapeutic effect on depression. However, this hypothesis should be confirmed in future studies. In this study, we observed a close correlation between KCNE1 and resting CD4+ memory T cells and M0 macrophages. However, these results have not yet been investigated and should be explored in future studies. As an ERS gene, protein disulphide isomerase family member 4 (PDIA4) is significantly correlated with the expression levels of inflammatory cytokines, a feature of MDD pathology (42, 43). Although the relationship between PDIA4 and MDD has not been reported, we speculate that this gene may be involved in pathological changes in MDD by influencing inflammatory factors. Staufen 1 (STAU1) is generally expressed in mammals, and its downregulation reduces the amplitude and frequency of small postsynaptic excitatory currents, suggesting that STAU1 is important for the processing or transport of dendritic mRNA (44). Interestingly, dendritic mRNA are critical for maintaining changes in functional connectivity, such as hippocampus-dependent learning and memory (45). Microsomal glutathione S-transferase 1 (MGST1) has been proven to be involved in the regulation of oxidative stress (46). Disruption of the insulin pathway in the brain is involved in the pathogenesis of depression (47). It has been reported that Src homology 2 domain containing (Shc1) is activated by the insulin receptor, which in turn activates Grb2 (48). The active Shc1/Grb2 complex stimulates intracellular signalling pathways, and its disturbance may contribute to spatial memory deficits in rats after water maze training (49). Meanwhile, antidepressant drugs may exert beneficial effects on the insulin receptor phosphorylation pathway through the Shc1/Grb2 complex (50), which further supports the potential use of Sch1 for depression drug development. However, direct evidence of the relationship between these genes and MDD pathogenesis has not been reported, and their functions in MDD require further exploration.

In the validation analysis, we confirmed that the model had good predictive power and that these seven genes could be used as diagnostic markers for MDD. Inflammatory processes are specifically involved in MDD, and the immune profiles of MDD and control groups were analysed. The results revealed that the infiltration level of M0 macrophages was higher in MDD samples than in control samples, which was also observed by Zhang et al. (51). A previous study indicated that a higher production of pro-inflammatory cytokines was detected in M0 macrophages in autologous sera from patients with MDD than in control samples (52). Resting CD4 memory T cells and resting dendritic cells also exhibited different levels of infiltration in the MDD and control groups. However, the mechanisms underlying these complex interactions between the diagnostic genes and immune cells require further investigation.

Furthermore, two ERS-related molecular subtypes for MDD patients were identified. SubA had higher expression value of ER scores than SubB, suggesting that patients in this subtype may be accompanied by a more pronounced activation of ERS. Moreover, the SubB subtype appeared to be immune-infiltrative because of its higher stromal and estimated scores than those of the SubA subtype. Evidence indicates that depression may damage the immune system and lead to immunosuppression (53). Thus, we speculated that patients in the SubA subtype may have higher severity of MDD. However, due to the lack of clinical information about patients, the correlation between subtypes and clinical characteristics is not evaluated, which is necessary to understand the clinical significance of subtypes. Importantly, we can confirm that ERS can regulate the immune microenvironment of MDD, thus affecting the course of the disease (54). In the future, the role of immune cell dynamics in different subtypes needs to be further explored.

Growing evidence suggests that drugs with major immune targets can ameliorate depressive symptoms (55). In addition, hub genes associated with SubB subtypes were screened by using WGCNA, such as NCF4, NCF2, CSF3R and FPR2. It has been showed that CSF3R is a cytokine that controls neutrophil expansion and differentiation, and can serve as a biomarker for neuromodulation; FPR2 knockdown may maintain hippocampal homeostasis by preventing depression-related neuronal damage (56, 57). The proteins encoded by NCF4 are cytoplasmic regulatory components of superoxide-producing phagocytic NADPH oxidase, which is a multicomponent enzyme system important for host defence (58). It primarily interacts with NCF2 and binds to NCF1 to form complexes that are transferred to membranes in response to cell stimulation (59). However, the functions of NCF4 and NCF2 have not yet been investigated in patients with MDD.

To the best of our knowledge, this is the first bioinformatic report describing ERS-related diagnostic genes and molecular subtypes of MDD. The present analysis has some unique advantages. Compared with previous diagnostic model construction (51), our analysis employs a larger number of datasets and identifies two molecular subtypes associated with ERS, which may provide a more comprehensive understanding of MDD. Notably, our findings clarify several diagnostic signatures and molecular subtypes from the perspective of ERS, which can provide a deeper understanding of the molecular heterogeneity of depression; meanwhile, it is helpful to assist psychiatrists to formulate accurate and individualised treatment schemes, thus reducing the burden of depression. Furthermore, previous study has indicated that antidepressant drugs or natural compounds can exert therapeutic effects via reducing ERS (17), and the selected biomarkers in this work could provide information for the development of more effective treatments.

Our study had some limitations. First, MDD is clinically heterogeneous; however, the disease type of the enrolled patients was not recorded in detail in the database. Therefore, this point was not considered in this analysis. In addition, the results were obtained through bioinformatics analysis and are still at the predictive stage, so there is insufficient experimental evidence to confirm our findings. Thus, further experiments are needed to confirm the specific mechanism of action of diagnostic genes in MDD.

## Conclusion

Taken together, our results established a diagnostic model based on seven ERS-related genes that exhibited robust and good estimation performance. Simultaneously, two ERS-associated molecular subtypes with different ER scores and immune characteristics were screened. Our findings provide a reliable model for MDD diagnosis and development of individualised treatment plans from an ERS perspective.

## Data availability statement

The original contributions presented in the study are included in the article/Supplementary material, further inquiries can be directed to the corresponding author.

## Author contributions

SH: conceptualization, data analysis, and draft preparation. YL, JS, and WL: data collection and analysis. CL: supervision, reviewing, and editing. All authors contributed to the article and approved the submitted version.

## Funding

This study was supported by The National Natural Science Foundation of China (Grant number: 82174248 and 81774209); Social Development Guiding Project of Science and Technology Department of Fujian Province (Grant number: 2019Y0039).

## Conflict of interest

The authors declare that the research was conducted in the absence of any commercial or financial relationships that could be construed as a potential conflict of interest.

## Publisher’s note

All claims expressed in this article are solely those of the authors and do not necessarily represent those of their affiliated organizations, or those of the publisher, the editors and the reviewers. Any product that may be evaluated in this article, or claim that may be made by its manufacturer, is not guaranteed or endorsed by the publisher.

## Footnotes

^1^ http://www.bioconductor.org/packages/release/bioc/html/sva.html

^2^ http://77.66.12.57/R-help/cor.test.html
